# 

**Published:** 1886-10

**Authors:** 


CONTENTS.

Vol. 33 No. 10

| | PAGE. |
|---|---|
| Dreams | 273 |
| Lanolin | 277 |
| Caves of Vultures | 279 |
| The Microscope | 281 |
| Pork as Food | 283 |
| Health and Exercise | 284 |
| Uses of Borax | 286 |
| Consumption Curable | 287 |
| Seven Hundred Album Verses | 287 |
| The Means of Contagion in Scarlet Fever | 288 |
| The Kindergarten | 288 |
| Comparative Tests of Infant Foods | 289 |
| Verification of a Dream After Forty Years | 290 |
| Excellent Bitters | 290 |
| Home Department | 291 |
| Miscellaneous Items | 294 |

| INDEX TO ADVERTISEMENTS OF HALF A PAGE AND OVER. | PAGE. |
|---|---|
| Fellows' Hypo-Phosphites | I |
| Lactated Food | II |
| Celerina | III |
| Lactopeptine | IV |
| Castoria | IV |
| Horsford's Acid Phosphate | V |
| Caulocorea | V |
| Bovinine | VI |
| Vaginal Injecting and Suction Syringe | VIII |
| Offer Extraordinary | IX |
| Medicated Suppositories | X |
| Dr. Buckland's Scotch Oats Essence | 2d page cover |
| Perforated Paper | 3d " " |
| Compound Oxygen | 4th " " |

